# Redefining the Role of Lymphotoxin Beta Receptor in the Maintenance of Lymphoid Organs and Immune Cell Homeostasis in Adulthood

**DOI:** 10.3389/fimmu.2021.712632

**Published:** 2021-07-15

**Authors:** Yajun Shou, Ekaterina Koroleva, Cody M. Spencer, Sergey A. Shein, Anna A. Korchagina, Kizil A. Yusoof, Raksha Parthasarathy, Elizabeth A. Leadbetter, Armen N. Akopian, Amanda R. Muñoz, Alexei V. Tumanov

**Affiliations:** ^1^ Department of Microbiology, Immunology and Molecular Genetics, University of Texas Health Science Center at San Antonio, San Antonio, TX, United States; ^2^ Department of Gastroenterology, Second Xiangya Hospital of Central South University, Changsha, China; ^3^ Trudeau Institute, Saranac Lake, NY, United States; ^4^ Department of Endodontics, University of Texas Health Science Center at San Antonio, San Antonio, TX, United States

**Keywords:** lymphotoxin, LTβR, lymphoid organs, FDCs, IgA, *Citrobacter rodentium*

## Abstract

Lymphotoxin beta receptor (LTβR) is a promising therapeutic target in autoimmune and infectious diseases as well as cancer. Mice with genetic inactivation of LTβR display multiple defects in development and organization of lymphoid organs, mucosal immune responses, IgA production and an autoimmune phenotype. As these defects are imprinted in embryogenesis and neonate stages, the impact of LTβR signaling in adulthood remains unclear. Here, to overcome developmental defects, we generated mice with inducible ubiquitous genetic inactivation of LTβR in adult mice (iLTβR^Δ/Δ^ mice) and redefined the role of LTβR signaling in organization of lymphoid organs, immune response to mucosal bacterial pathogen, IgA production and autoimmunity. In spleen, postnatal LTβR signaling is required for development of B cell follicles, follicular dendritic cells (FDCs), recruitment of neutrophils and maintenance of the marginal zone. Lymph nodes of iLTβR^Δ/Δ^ mice were reduced in size, lacked FDCs, and had disorganized subcapsular sinus macrophages. Peyer`s patches were smaller in size and numbers, and displayed reduced FDCs. The number of isolated lymphoid follicles in small intestine and colon were also reduced. In contrast to LTβR^-/-^ mice, iLTβR^Δ/Δ^ mice displayed normal thymus structure and did not develop signs of systemic inflammation and autoimmunity. Further, our results suggest that LTβR signaling in adulthood is required for homeostasis of neutrophils, NK, and iNKT cells, but is dispensable for the maintenance of polyclonal IgA production. However, iLTβR^Δ/Δ^ mice exhibited an increased sensitivity to *C. rodentium* infection and failed to develop pathogen-specific IgA responses. Collectively, our study uncovers new insights of LTβR signaling in adulthood for the maintenance of lymphoid organs, neutrophils, NK and iNKT cells, and IgA production in response to mucosal bacterial pathogen.

## Introduction

Lymphotoxin beta receptor (LTβR) belongs to the tumor necrosis factor receptor superfamily (TNFR) and is known as a key regulator of lymphoid organogenesis and inflammation (1–4). Therapeutic strategies for inhibition or stimulation of LTβR signaling are currently in development for treatment of inflammatory and infectious diseases as well as cancer (5–7). However, the impact of LTβR inactivation in adulthood remains incompletely understood.

LTβR is primarily expressed by epithelial cells, stromal cells, dendritic cells (DCs), and macrophages (Mph), but is absent on lymphocytes (1, 8, 9). LTβR interacts with two ligands: membrane heterotrimeric lymphotoxin LTα1β2 (LT) and homotrimeric LIGHT, both expressed predominantly by lymphoid cells (1, 9, 10). In contrast to LTα1β2 heterotrimer, LTα3 homotrimer interacts with TNFR1 and TNFR2 but not LTβR (1, 9). Therefore, LTα^-/-^ mice share defects in both LTβR and TNFR signaling. LTα^-/-^ and LTβR^-/-^ mice lack all lymph nodes (LNs), Peyer`s patches (PPs), isolated lymphoid follicles (ILFs) and cryptopatches in the gut. Additionally, these mice display disorganized spleen and thymus structure as well as defects in homeostasis of DCs (11, 12), NK cells (13), and NKT cells (14, 15). Furthermore, LTα^-/-^ and LTβR^-/-^ mice are known to have impaired IgA production (16–18). Membrane-bound LTα1β2 is the critical LTβR ligand which is required for lymphoid organogenesis (1, 2, 19). LT expressed by group 3 innate lymphoid cells (ILC3s) is required for the development of lymph nodes and Peyer`s patches during embryogenesis (17, 20), whereas LT expression by B cells is critical for the maintenance of spleen structure in adulthood (21, 22). Although LIGHT^-/-^ mice do not exhibit defects in development and structure of lymphoid organs, LIGHT can also contribute to the maintenance of lymphoid organs, immune response to pathogens and autoimmunity (23–26).

LTβR signaling can activate both canonical and alternative NF-kB signaling to induce various proinflammatory chemokines and cytokines (1, 9, 27). Paradoxically, LTβR^-/-^ mice exhibit an autoimmune phenotype which includes splenomegaly, autoantibody production and systemic inflammation with increased neutrophil numbers in spleen and multiple lymphocytic lymphoid infiltrates in non-lymphoid organs (28–30). Several explanations for an autoimmune phenotype in these mice have been suggested, including lack of lymph nodes and follicular dendritic cells (FDCs) (31, 32), impaired thymus structure and central tolerance (29, 33–35), and altered microbiota composition (30, 36). Since these defects are imprinted during embryogenesis or early neonatal stages, the role of LTβR in lymphoid tissue maintenance, autoimmunity, homeostasis of innate immune cells, IgA production and immune responses in adulthood remain unclear.

Biochemical inhibition of LTβR signaling with LTβR-Ig, a soluble decoy protein containing the extracellular portion of LTβR fused with the Fc portion of IgG, has proven to be a valuable tool for deciphering the role of LTβR signaling in adulthood (5). LTβR-Ig treatment partially impaired the structure of secondary lymphoid organs, and showed therapeutic efficacy in various models of inflammatory diseases (5). However, interpretation of LTβR-Ig effects *in vivo* is complicated, as it blocks both membrane LT and LIGHT ligands, which are known to play distinct and overlapping functions due to LIGHT binding to another receptor, HVEM (1, 26, 37, 38). Additionally, repeated administration of LTβR-Ig may induce side effects due to anti-drug antibody formation and modulation of Fc-linked receptors (39, 40).

In this study, to define the role of LTβR signaling in adulthood independent from LTβR signaling during embryogenesis and neonate stages, we generated mice with inducible ubiquitous genetic inactivation of LTβR in adult mice (iLTβR^Δ/Δ^ mice). Our results demonstrate that LTβR signaling in adulthood is critical for the maintenance of spleen, lymph nodes, gut-associated lymphoid organs; homeostasis of neutrophils, NK and iNKT cells; and specific IgA antibody responses against mucosal pathogen *Citrobacter rodentium;* but is dispensable for the maintenance of polyclonal IgA production and autoimmunity.

## Materials and Methods

### Mice

LTβR^-/-^ and LTβR^fl/fl^ mice were described previously (41). R26-CreERT2 mice (stock #008463) (42) and MRL/MpJ-*Fas^lpr^*/J (MRL mice) (stock #000485) were purchased from the Jackson Laboratory. Mice with inducible LTβR deletion (iLTβR^Δ/Δ^ mice) were generated by crossing LTβR^fl/fl^ mice with R26-CreERT2 mice. 6-8 week old adult mice were treated with 5mg tamoxifen (TAM) in 100μl of corn oil containing 10% ethanol by oral gavage for 4 consecutive days to generate stable deletion of *Ltbr* gene (iLTβR^Δ/Δ^ mice). Depending for experiment, two different types of experimental controls were used: Cre-positive LTβR^fl/fl^ littermates treated with corn oil containing 10% ethanol or Cre-negative LTβR^fl/fl^ littermates treated with TAM. We did not find any difference between these two control groups (data not shown). Both sexes were used for experiments. Mice were used for experiments one month after TAM treatment, unless specified. Animals were housed under specific-pathogen-free conditions in accordance with National Institutes of Health guidelines. All experimental animal procedures were approved by the Institutional Animal Care and Use Committee of University of Texas Health Science Center San Antonio.

### Preparation of Colonic Lamina Propria Lymphocytes

Lamina propria lymphocytes (LPL) were isolated as described previously (41). Briefly, the cecum and colon were cut open and rinsed twice in PBS to remove fecal material. Colon and cecum pieces were incubated in complete medium (RPMI-40 supplemented with 3% FBS, 1 mM penicillin-streptomycin) containing 2 mM EDTA for 20 min at 37°C with slow rotation (100 rpm) to remove epithelial cells. Remaining tissue pieces were digested in serum-free RPMI-40 containing 200 μg/ml Liberase TM (Roche) and 0.05% DNase I (Sigma) for 40 min at 37°C with shaking at 100 rpm. The digested tissue was passed through a mesh screen, washed with PBS containing 3% FBS and separated by 80/40% Percoll gradient (GE Healthcare).

### Flow Cytometry

Single cell suspensions were prepared from spleen, mesenteric lymph nodes (MLN), inguinal lymph nodes (ILN), PP, and thymus using 70μm nylon cell strainers. Intrahepatic lymphocytes were isolated using steel mesh (150 μm). Cells were resuspended in 40% isotonic Percoll (GE Healthcare) and centrifuged at 930g at room temperature for 20 min. Red blood cells were lyzed with ACK solution before antibody staining. The cells were preincubated for 20 min with anti-CD16/32 Fc blocking antibody (2.4G2) and with Zombie UV™ Fixable Viability Dye (Biolegend). Cell surface staining for flow cytometry was done using a combination of the following antibodies: anti-CD45.2 (104), anti-CD3 (17A2), anti-B220 (RA3-6B2), anti-CD4 (GK1.5), anti-CD8 (53-6.7), anti-NK1.1 (PK136), anti-TCRβ (H57-597), anti-GL-7 (GL7), anti-IgG (A85-1), anti-IgA (mA-6E1), anti-IgM (RMM-1), anti-CD138 (281-2), anti-CD19 (ID3), anti-CD11b (M1/70), anti-Ly6G (IA8). *i*NKT cells were identified using FITC conjugated mCD1d−αGalCer tetramers (PBS57, NIH tetramer Core). All antibodies were purchased from BD Biosciences, Biolegend or eBioscience. Flow cytometry analysis was performed on a BD FACSCelesta and analyzed using FlowJo 10 software.

### Real-Time RT-PCR Analysis

RNA from murine tissue was isolated using the E.Z.N.A. Total RNA kit I (Omega Bio-tek). cDNA synthesis and real-time RT-PCR was performed as described previously (41) using Power SYBR Green master mix (Applied Biosystems). Relative mRNA expression of target genes was determined using the comparative 2^−ΔΔCt^ method. Primers used are listed in **Supplementary Table 1**.

### Analysis of LTβR Gene Deletion

To assess LTβR deletion efficiency, tissue was homogenized in 800μl lysis buffer (100mM Tris-HCL, 5mM EDTA, 0.2% SDS, 200mM NaCl). 4μl of 20mg/ml proteinase K was then added to each samples and samples were incubated at 55°C on a shaker overnight. Tubes were vortexed and centrifuged for 15 minutes at full speed in a microcentrifuge. Supernatants were transferred to new 1.5mL tubes and DNA precipitated with 500μl of isopropanol. The DNA pellets were washed with 70% ethanol and resuspended in 70μl TE buffer (10mM Tris, 1mM EDTA, pH 7.5). DNA deletion of LTβR was analyzed by PCR using primers: 351- CAGTGGCTCCAAGTGGCTTG, 352-GCAAACCGTGTCTTGGCTGC, and 441- ACAGGGCAGACATTAGGGTTCC as described (41). LTβR deletion: 360 bp (primers 352-441), LTβR flox: 376 bp (primers 351-352).

### Histology and Immunohistochemistry

Frozen sections of spleen, MLN, PPs were stained with antibodies: B220-PB (RA3-6B2, Biolegend), CD3e-PE (145-2C11, BD Biosciences), CD21/CD35-FITC (7E9, Biolegend), ER-TR7-AF647 (ER-TR7, Novus Bio), MAdCAM-1-PE (MECA-367, Biolegend), SIGNR1-APC (22D1, eBioscience), CD169-AF488 (3D6.112, Biolegend). Sections were analyzed using Zeiss LSM710 confocal microscope. Image processing was done using Zeiss software. Analysis of mean fluorescent intensity (MFI) was done using Image J, as described (43). Data is presented as normalized MFI. Small intestine (SI), colon, spleen, liver, thymus, and lung tissues were fixed in 10% neutral buffered formalin and analyzed by hematoxylin and eosin (H&E) staining. Spleens were also labeled with CD35 (CR1, 8C12, BD Biosciences) and MAdCAM-1 (MECA-367, Biolegend) followed by horseradish peroxidase (HRP) conjugated secondary antibody followed by AEC Vector kit (Vector Labs) to assess spleen microarchitecture as described (22).

### *C. rodentium* Infection and Assessment of Bacterial Burden

*C. rodentium* infection and analysis of bacterial burdens was done as described (44). Briefly, mice were infected with *C. rodentium* strain DBS100 (ATCC 51459) by oral gavage with 2*10^9^ colony-forming units (CFUs) in 0.2mL of PBS. Tissue samples were homogenized in PBS, serially diluted, and plated on MacConkey agar plates. CFUs were counted after incubation at 37°C for 18-24 hours.

### ELISA

For the analysis of total IgA, IgG, and IgM in sera and feces, 96 well plates (Immulon 4 HBX) were first coated with anti-mouse IgA (1:500), IgG (1:1000), or IgM (1:1000) diluted in 1x carbonate-bicarbonate buffer at room temperature overnight. Plates were then washed 3 times with PBS containing 0.1% tween 20 (PBST). Serially diluted serum or fecal extracts were added and incubated at 25°C for 1 hour. Plates were washed, and HRP-conjugated goat anti-mouse IgG, IgA, or IgM antibodies (Southern Biotechnology Associates, Inc.) were added and incubated for 1h at 37°C. Plates were developed using ABTS (1-Step™ ABTS Substrate Solution (ThermoFisher) and OD410 values obtained on a BioTek SynergyHT plate reader. Analysis of *C. rodentium*-specific immunoglobulin (IgA, IgG and IgM) levels in serum or fecal samples was performed as described (44). Anti-dsDNA antibody detection was done on serum samples from naïve mice 2-4 months after TAM treatment as previously described (45). Briefly, plates were precoated with methylated BSA overnight (5μg/mL) at 4°C overnight. Plates were then washed with PBST and coated with 50μg/mL of calf thymus DNA (Sigma D-4522) at 4°C overnight, blocked with BSA (5μg/mL) and diluted serum added for 2h at room temperature followed by secondary HRP-conjugated antibody and TMB substrate detection.

### Statistical Analysis

All statistics were determined using GraphPad Prism software (v 9). Statistical significance was determined using one-way ANOVA or two-way ANOVA with Tukey’s multiple comparison test, Mann-Whitney test, Kruskal Wallis test with Dunn’s correction, or unpaired Student’s *t*-test as appropriate. Survival was assessed using the Log-rank (Mantel-Cox) and Gehan-Breslow-Wilcoxon tests. Not significant, p > 0.05 (ns); p< 0.05 (*); p< 0.01 (**); p< 0.001 (***); p< 0.0001 (****).

## Results

### Inducible Genetic Inactivation of LTβR in Adulthood

To clearly define the role of LTβR signaling in the adulthood, independent from the role of LTβR during embryogenesis and neonate stages, we utilized a well-established genetic approach to induce ubiquitous gene deletion. To do this, we crossed LTβR^fl/fl^ mice (41) with transgenic mice expressing *Cre* recombinase linked to estrogen receptor-T2 (Cre-ERT2) (42), generating mice with inducible LTβR gene inactivation (iLTβR^Δ/Δ^ mice). Tamoxifen (TAM) administration permits *Cre* release to the nucleus and induces recombination of target LoxP sites. Adult iLTβR^Δ/Δ^ mice were treated with 5 mg of tamoxifen (TAM) in 100μl of corn oil containing 10% ethanol by oral gavage for 4 consecutive days. To define the efficacy of LTβR inactivation in mice following TAM treatment, we analyzed LTβR expression by qPCR from tissues collected from iLTβR^Δ/Δ^ and control LTβR^fl/fl^ mice one month after TAM treatment (**Figure S1A**). In tissues with high epithelial/mesenchymal cell populations (colon, liver, and kidney), the LTβR mRNA downregulation was over 90% (**Figure S1A**). Notably, LTβR expression in the colon and liver was decreased 100-fold in iLTβR^Δ/Δ^ mice following oral or intraperitoneal TAM treatment (**Figure S1A, B**). High efficacy of LTβR mRNA inhibition was also detected in lymphoid organs: mesenteric LN (MLN), inguinal LN (ILN), PPs and spleen (**Figure S1A**). Overall, we found the highest reduction of LTβR expression in organs, which have large populations of LTβR expressing cells (epithelial cells, stromal cells, DCs and Mph). To confirm LTβR gene deletion, we assessed genomic LTβR DNA levels in colons from iLTβR^Δ/Δ^ mice after TAM treatment (**Figure S1C**). PCR analysis with primers flanking loxP sites showed effective deletion of LTβR promoter and the first two exons (**Figure S1C, D** **).** Combined, the mRNA and genomic DNA analyses demonstrate an effective inactivation of LTβR in adult mice following TAM treatment.

### LTβR Signaling in Adulthood Is Required for the Maintenance of LNs, PP and ILFs

**Figure 1:**
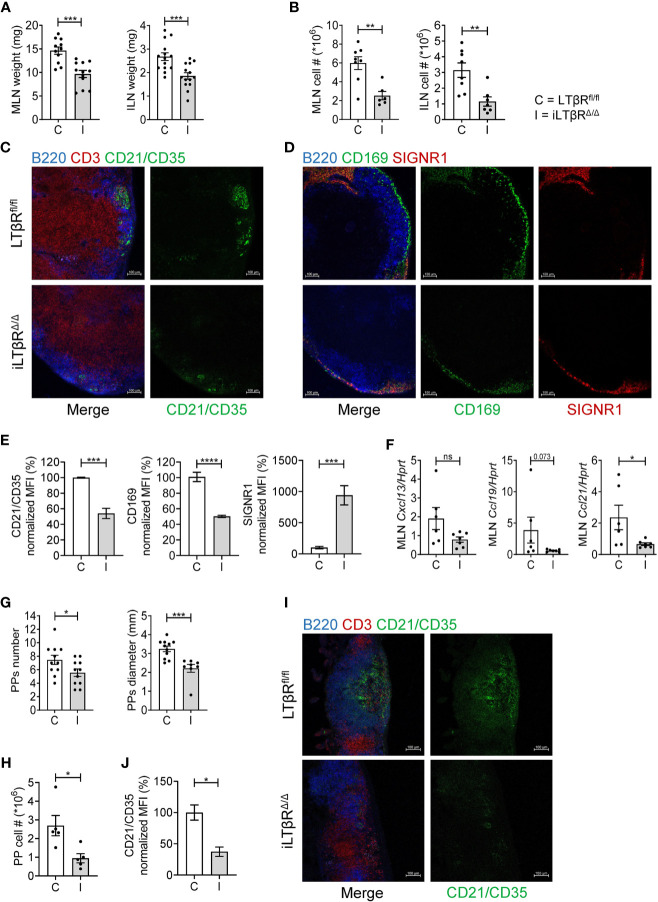
LTβR signaling in adulthood is required for the maintenance of LNs and PPs. LTβR^fl/fl^ (C) and iLTβR^Δ/Δ^ (I) mice were treated with TAM by oral gavage one month before analysis. **(A, B)** MLN and ILN weights and total cell numbers. ILN weights are the combined weight for both ILNs isolated from each mouse. Per group, n=8-12 for MLN and n=7-14 for ILN. **(C)** Representative confocal images of frozen MLN sections stained with antibodies against B220 (blue), CD3 (red) and CD21/CD35 (green). **(D)** Representative confocal images of MLN sections stained with antibodies against B220 (blue), CD169 (green) and SIGNR1 (red). **(E)** Quantification of FDCs and SCS macrophages from panels C and D. SIGNR1 signal was quantified in the subcapsular sinus region. n=4 for each group. **(F)** CXCL13, CCL19 and CCL21 expression in MLN of LTβR^fl/fl^ or iLTβR^Δ/Δ^ mice determined by qPCR (n=6-7). **(G)** Number and diameter of PPs. n=11 mice per group. **(H)** Number of cells isolated from PPs of LTβR^fl/fl^ or iLTβR^Δ/Δ^ mice. **(I)** Representative confocal micrographs of frozen PP sections. n=5 mice per group. **(J)** Quantification of CR21/35 MFI within individual PPs. For panels I-J, n=3 per group. Scale bars are 100μm for all images. Collective data from 4 experiments is shown for **(A, B, G)**. For **(C–F, I, J)**, data from 1 of 2 similar experiments are shown. For **(H)**, collective data from 2 of 3 experiments is shown. Significance was determined using Mann-Whitney or Unpaired t-test. Data shown are means ± SEM. Bars show the mean, symbols represent individual mice. Not significant (ns, p >0.05), *p < 0.05, **p < 0.01, ***p < 0.001, ****p < 0.0001.

LTβR^-/-^ mice fail to develop LNs and PPs due to lack of LTβR signaling in embryogenesis (4, 28). We found that despite normal development of LNs, iLTβR^Δ/Δ^ mice had smaller LNs compared to controls (**Figure 1A**). Consistent with the smaller LN size, total cell numbers in LNs of iLTβR^Δ/Δ^ mice were reduced (**Figure 1B**). These results suggest that LTβR signaling in adulthood contributes to the migration of naïve lymphocytes to the lymph nodes. Flow cytometry analysis confirmed reduction of B, CD4^+^ T and CD8^+^ T cells in the LNs of iLTβR^Δ/Δ^ mice compared to WT mice (**Figures S2A**, **S3A, B**). In MLNs, B and CD4^+^ T cell proportions were not changed while CD8^+^ T cells were reduced in iLTβR^Δ/Δ^ mice (**Figure S3A**). ILN cell proportions in iLTβR^Δ/Δ^ mice were differentially affected as B cells were decreased, CD4^+^ T cells were increased and CD8^+^ T cells populations were comparable to WT mice (**Figure S3B**). To define the impact of genetic LTβR inactivation on LN microarchitecture in adulthood, we next analyzed B cell follicles, follicular dendritic cells (FDCs) and sinus macrophages in the MLN of iLTβR^Δ/Δ^ mice (**Figures 1C–E**). FDCs play an important role in germinal center development by presenting antigen to B cells (46, 47). In iLTβR^Δ/Δ^ mice, B cell areas were disorganized and FDCs were significantly diminished (**Figures 1C, E**). Within LNs, sinus macrophages are divided into two subgroups, CD169^+^SIGNR1^-^ subcapsular sinus (SCS) macrophages and CD169^lo^SIGNR1**^+^** medullary sinus macrophages (48). Although both populations of sinus macrophages can capture antigen, their functions differ and are dependent on their location within the LN (48, 49). We found a reduced number of CD169**^+^**SCS macrophages in MLN of iLTβR^Δ/Δ^ mice. Interestingly, these macrophages co-expressed SIGNR1, which is normally expressed by medullary sinus macrophages (**Figures 1D, E**).

LTβR signaling controls the migration of B and T cells to lymphoid tissues *via* production of homeostatic CXCL13, CCL19, CCL21 chemokines and adhesion molecules (31, 50). Consistently, the expression of CCL21 was reduced in the MLN of iLTβR^Δ/Δ^ mice (**Figure 1F**). Although not significant, CCL19 demonstrated a declining trend (**Figure 1F**). CXCL13 expression was slightly reduced, although not significantly (**Figure 1F**). Together, these results demonstrate that LTβR signaling in adulthood is required for LN cellularity, production of homeostatic chemokines, as well as maintenance of FDCs and SCS macrophages.

We next sought to characterize the effects of inducible genetic LTβR inactivation in adulthood on the microarchitecture of PPs. Compared to WT mice, iLTβR^Δ/Δ^ mice displayed fewer and smaller PPs (**Figure 1G** and **Figure S4A**). The smaller number and size of PPs in iLTβR^Δ/Δ^ mice corresponded to a reduction in the number of cells isolated from PPs though the proportion of B, CD4^+^ T and CD8^+^ T cells was not changed compared to controls (**Figure 1H** and **Figure S3C**). Immunohistochemical analysis of PP structure revealed fewer FDCs and poorly defined B and T cell areas (**Figures 1I, J**) suggesting that active LTβR signaling in adulthood is required for the maintenance of PPs.

In contrast to PPs which develop during embryogenesis and require LTβR signaling in stromal cells (3), ILFs develop postnatally under influence of exogenous stimuli (51). The requirements of LTβR signaling for the development of ILFs during embryogenesis and adulthood are distinct. Although prenatal LTβR-Ig treatment results in accelerated formation of ILFs (52), postnatal LTβR-Ig treatment leads to elimination of ILFs (53). The number of ILFs in the small intestine (SI) and colon were reduced in iLTβR^Δ/Δ^ mice compared to control mice (**Figures S4A, B**). Our results corroborate previous findings and demonstrate that LTβR signaling in adulthood is required for the maintenance of LNs and PPs and formation of ILFs in the SI and colon.

### LTβR Signaling Is Required to Maintain Spleen Microarchitecture in Adulthood

The spleen is characterized by its distinctive white pulp (WP) and red pulp (RP) areas (46, 47). Both WP and RP areas are permeated by cells which play specialized roles in splenic homeostasis, architecture, and in immune protection (46, 47). In the marginal zone (MZ), which separates the WP and RP, antigens are captured for presentation to maturing B and T cells within the WP (46, 47). LTβR^-/-^ mice are known to have disorganized spleen structure evidenced by lack of MZ and clearly defined RP and WP areas [**Figure S4A**, and (28)]. Unlike LTβR^-/-^ mice, iLTβR^Δ/Δ^ mice had less disorganized spleens though their structure was notably impaired when compared to WT mice (**Figure S4A**). Although the WP size was similar in iLTβR^Δ/Δ^ mice and control mice, the MZ was less defined (**Figure S4A** **)**. LTβR^-/-^ mice are known to develop splenomegaly due to increased numbers of splenocytes and neutrophils (30, 54). The weight of spleens from iLTβR^Δ/Δ^ mice was comparable to WT mice (**Figure S4C**) and the proportion of B and CD4^+^ T cells was not changed in comparison to WT or LTβR^-/-^ mice (**Figure S3D**). CD8^+^ T cells were reduced in LTβR^-/-^ but not in iLTβR^Δ/Δ^ mice when compared to WT mice. These results suggest that genetic inactivation of LTβR in adulthood does not induce the development of splenomegaly.

**Figure 2:**
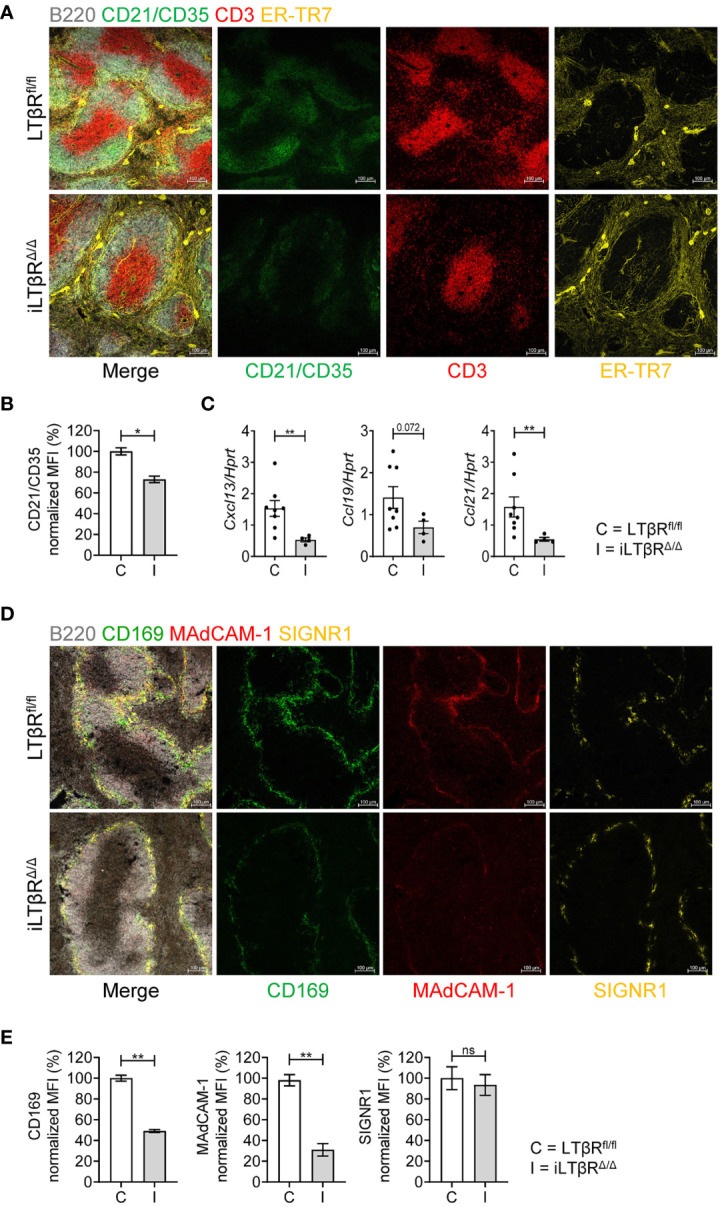
Impact of inducible LTβR inactivation in adulthood on spleen microarchitecture. LTβR^fl/fl^ (C) and iLTβR^Δ/Δ^ (I) mice were treated with TAM by oral gavage one month before analysis. **(A)** Representative confocal images of frozen spleen sections stained with antibodies against B220 (white), CD21/CD35 (green), CD3 (red) and ER-TR-7 (yellow). **(B)** Quantification of FDCs. n=4 per group. **(C)** Expression of splenic CXCL13, CCL19, and CCL21 mRNA determined by qPCR (n=4-8). **(D)** Representative confocal images of frozen spleen sections stained with antibodies against B220 (white), CD169 (green), MAdCAM-1 (red) and SIGNR1 (yellow) to analyze the marginal zone structure. **(E)** Quantification of CD169, MAdCAM-1 and SIGNR1 expressing cells. n=4 per group. Scale bars = 100μm for all images. Data shown are means ± SEM. Bars show the mean, symbols represent individual mice. Data is from one of two experiments with similar results. Significance was determined using Mann-Whitney or Unpaired t-test. Not significant (ns, p > 0.05), *p < 0.05, **p < 0.01.

To assess the spleen microarchitecture of iLTβR^Δ/Δ^ mice, we stained frozen spleen sections with CD21/CD35, CD3, B220, and ER-TR-7 antibodies (**Figures 2A**, **S4D**). Notably, organized B cell follicles were absent, while B cells formed a ring-like structures around T cells areas (**Figure 2A**). Further, FDC numbers were strongly reduced in spleen of iLTβR^Δ/Δ^ mice (**Figures 2A, B**, **S4D**). ER-TR7^+^ fibroblastic reticular networks were less organized in iLTβR^Δ/Δ^ mice compared to WT mice (**Figure 2A**). However, the degree of spleen disruption in iLTβR^Δ/Δ^ mice was not as strong as in LTβR^-/-^ mice which displayed reduced WP area, mixed T and B cell areas, complete loss of FDCs and the marginal zone (**Figure S4A** and data not shown). Consistent with reduced FDCs and less organized stromal components, iLTβR^Δ/Δ^ mice had reduced expression of homeostatic chemokines CXCL13, CCL21, and CCL19 (**Figure 2C**).

To define the role of LTβR signaling for the maintenance of the marginal zone in adulthood, we stained spleens of iLTβR^Δ/Δ^ mice with CD169, MAdCAM-1^+^ and SIGNR1 antibodies (**Figure 2D**). The number of MAdCAM-1^+^ and CD169^+^ cells was dramatically reduced, whereas the number of SIGNR1^+^ macrophages was less affected in spleen of iLTβR^Δ/Δ^ mice (**Figures 2D, E** and **Figure S4D**). These results suggest that LTβR signaling in adulthood is required for the maintenance of spleen microarchitecture.

### iLTβR^Δ/Δ^ Mice Do Not Develop Autoimmunity and Systemic Inflammation

**Figure 3:**
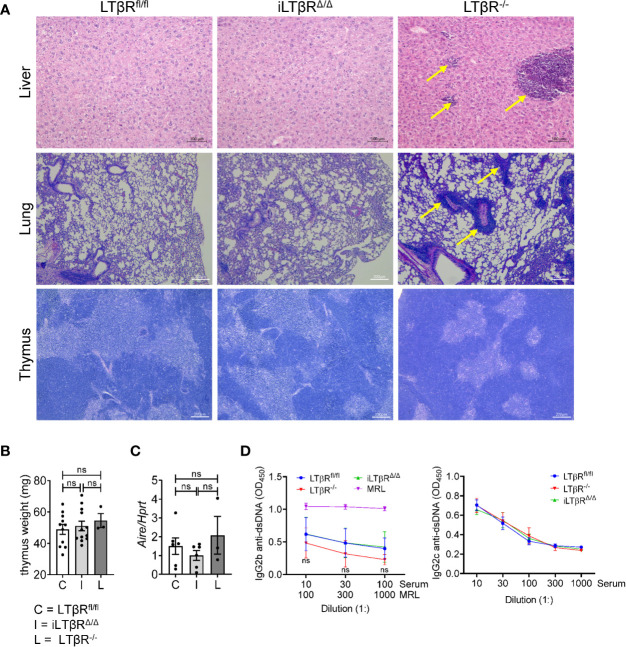
iLTβR^Δ/Δ^ mice do not develop autoimmunity. **(A)** Representative H&E images of liver (scale bars 100μm), lung (scale bars 200μm), and thymus (scale bars 200μm) from iLTβR^Δ/Δ^ mice at one (liver and lung) or two (thymus) months post TAM treatment. Oil treated LTβR^fl/fl^ (C) and untreated LTβR^-/-^ (L) mice were used as controls. Arrows indicate lymphoid infiltrates in LTβR^-/-^ mice. N=5 for all groups. **(B)** Thymus weight. n=3-12. **(C)** Expression of *Aire* mRNA in thymus determined by qPCR (n=3-6 per group). **(D)** Analysis of anti-dsDNA antibodies by ELISA in mice 2-4 months after TAM treatment (n=20 for WT, n=14 for iLTβR^Δ/Δ^, n=9 for LTβR^-/-^). Data shown are means ± SEM. Bars show the mean, symbols represent individual mice. Data was derived from 1-2 experiments for all panels. Significance was determined using one-way ANOVA with Tukey’s correction. Not significant (ns, p>0.05).

Splenomegaly is often associated with systemic inflammation and autoimmunity (30, 55). To define the role of LTβR signaling in adulthood on autoimmunity and systemic inflammation, we analyzed the liver, lung, and thymus of iLTβR^Δ/Δ^ mice for tissue abnormalities or key autoimmune indicators (**Figure 3**). Histological analysis of livers showed considerable perivascular lymphocytic infiltration in LTβR^-/-^ but not in iLTβR^Δ/Δ^ mice relative to that of age-matched WT controls (**Figure 3A**). The lungs of LTβR^-/-^ mice also displayed an enhanced pattern of perivascular inflammation that was not detected in iLTβR^Δ/Δ^ mice (**Figure 3A**). These results suggest that formation of lymphocytic infiltrates observed in adult LTβR^-/-^ mice depends on LTβR inactivation during embryogenesis or neonate stages.

We next analyzed thymus organization, as impaired negative selection of lymphocytes due to defects in thymus structure was attributed to autoimmunity in LTβR^-/-^ mice (29, 56, 57). Several studies demonstrated the role of LTβR signaling in controlling thymic epithelial cells, endothelial cells and stromal cells (29, 33, 34, 56–58). Our analysis revealed that thymus structure of iLTβR^Δ/Δ^ mice was not impaired (**Figure 3A**). In contrast, thymic medulla of LTβR^-/-^ mice was smaller and disorganized (**Figure 3A**). Thymus weight of iLTβR^Δ/Δ^ and LTβR^-/-^ mice were normal (**Figure 3B**). Consistent with the lack of thymus impairment in iLTβR^Δ/Δ^ mice, expression levels of *Aire*, a transcriptional regulator of autoimmunity, were not reduced (**Figure 3C**). The proportion of B, CD8^+^ T, and CD4^+^CD8^+^ T cells were also not affected (**Figures S2C**, **S3F** **)**. However, an increase in the proportion of thymic CD4^+^ T cells compared to WT mice was observed in iLTβR^Δ/Δ^ and LTβR^-/-^ mice was observed compared to WT mice (**Figure S3F**). These results are consistent with previous studies conducted in LTβR^-/-^ mice (33, 58). Collectively, this data suggest that inducible inactivation of LTβR in adult mice does not impair thymus organization. Furthermore, serological analysis of dsDNA autoantibody levels, a hallmark of autoimmune disease, demonstrated a lack of autoantibodies in iLTβR^Δ/Δ^ mice compared to control MRL mice (**Figure 3D**). Additionally, we did not find changes in the proportions of B, CD8^+^ T, and CD4^+^ T cells in the blood of iLTβR^Δ/Δ^ and LTβR^-/-^ mice compared to WT mice (**Figure S3E**). Thus, our results suggest that inactivation of LTβR signaling in adulthood does not result in autoimmunity and systemic inflammation.

### LTβR Signaling Controls Homeostasis of Neutrophils, NK, and iNKT Cells in Adult Mice

**Figure 4:**
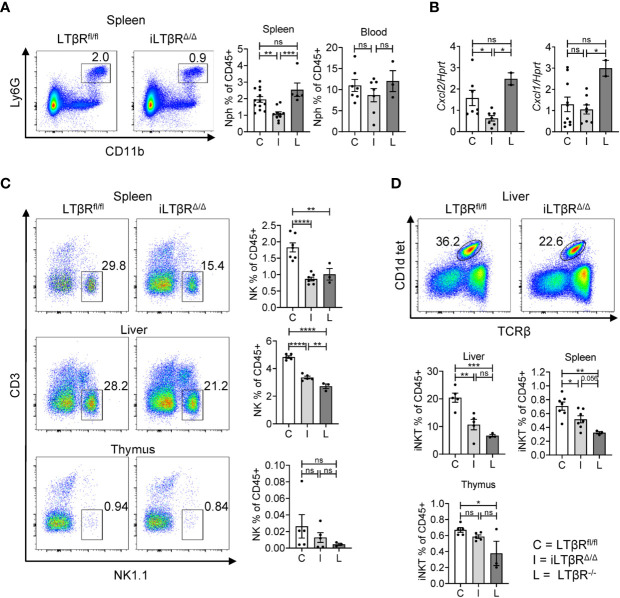
Impact of LTβR signaling in adulthood on homeostasis of neutrophils, NK, and iNKT cells. LTβR^fl/fl^ (C) and iLTβR^Δ/Δ^ (I) mice were treated by oral gavage with oil or TAM one month before analysis. **(A)** Representative flow cytometry plots of neutrophils (Nph) in the spleens of iLTβR^Δ/Δ^ and LTβR^fl/fl^ mice. Graphs show % of Ly6G^+^ Lin^-^CD11b^+^ neutrophils in CD45^+^ gate for spleens and blood. **(B)** Expression of CXCL1 and CXCL2 in the spleen measured by qPCR. For panels **(A, B)**, data shown is combined from 3 experiments. N=2-13 per genotype. **(C)** Representative flow cytometry plots of NK1.1^+^ NK cells in the spleen, liver, and thymus from iLTβR^Δ/Δ^ and LTβR^fl/fl^ mice. Right panels indicate % of NK cell frequencies in the CD45^+^ populations from spleen, liver, and thymus. **(D)** Representative flow cytometry plots of CD1d tet^+^TCRβ^+^ iNKT cells in the liver. Graphs show % of iNKT cell frequencies in the CD45^+^ populations of liver, thymus, and spleen. For panels **(C, D)**, data shown is from two of three independent experiments. Significance was determined using one-way ANOVA with Tukey’s correction for multiple comparisons. All gating strategies are defined in **Figure S2**. Data shown are means ± SEM. Bars show the mean, symbols represent individual mice. Not significant (ns, p > 0.05), *p < 0.05, **p < 0.01, ***p < 0.001, ****p < 0.0001.

Defects in homeostasis of neutrophils, NK, and NKT cells have been described in LTβR^-/-^ mice (13–15, 30, 59–61). However, the role of LTβR signaling in homeostasis of these cells in adult mice is not clear, as defects in development of lymphoid organs and systemic inflammation in LTβR^-/-^ mice can affect these cells. To clarify this, we analyzed neutrophils, NK, and iNKT cells in iLTβR^Δ/Δ^ mice by flow cytometry (**Figures 4** and **S2**). A previous study detected increased numbers of neutrophils in the spleens of adult LTβR^-/-^ mice which was dependent on microbiota (30). Increased neutrophil numbers were also detected in the blood of adult BALB/C mice treated weekly with LTβR-Ig for four weeks (31). Surprisingly, we found reduced frequencies of neutrophils in the spleens of iLTβR^Δ/Δ^ mice, but not in blood (**Figure 4A**), but not in blood (**Figure 4A**) compared to LTβR^fl/fl^ and LTβR^-/-^ mice. As recruitment of neutrophils is mainly dependent on CXCL2 and CXCL1 chemokines (62, 63), we measured expression of these chemokines in the spleens of iLTβR^Δ/Δ^ mice. Expression of CXCL2 was reduced in iLTβR^Δ/Δ^ mice compared to control or LTβR^-/-^ mice (**Figure 4B**). However, expression of CXCL1 was not changed in iLTβR^Δ/Δ^ mice compared to controls though it was significantly lower than levels observed in LTβR^-/-^ mice (**Figure 4B**). These results suggest that active LTβR signaling in adulthood is required for recruitment of neutrophils to the spleen.

Consistent with previous studies in LTβR^-/-^ mice and mice treated with LTβR-Ig (13, 61), our analysis revealed reduced frequencies of NK cells in the spleens and livers of iLTβR^Δ/Δ^ and LTβR^-/-^ mice compared to LTβR^fl/fl^ mice (**Figure 4C**). No difference was observed in spleen NK cell frequencies between LTβR^-/-^ and iLTβR^Δ/Δ^ mice, although there was an increase in liver NK cell frequencies from iLTβR^Δ/Δ^ mice compared to LTβR^-/-^ mice (**Figure 4C**). NK cell frequencies were not significantly reduced in the thymuses of iLTβR^Δ/Δ^ mice (**Figure 4C**). Additional analysis of iNKT cells (CD1d-tet^+^TCRβ^+^NK1.1^+^) revealed no difference in iNKT cell proportions in the thymuses of iLTβR^Δ/Δ^ mice compared to LTβR^fl/fl^ control mice though iNKT cell frequencies were reduced in LTβR^-/-^ mice compared to controls (**Figure 4D**). In contrast, iNKT cell frequencies were reduced in the livers and spleens of LTβR^-/-^ and iLTβR^Δ/Δ^ mice compared to WT mice (**Figure 4D**). These results suggest that LTβR signaling supports the migration of iNKT cells to the liver and spleen in adulthood but is dispensable for iNKT cell development in the thymus.

### LTβR Signaling Is Not Required for the Maintenance of IgA in Adulthood

**Figure 5:**
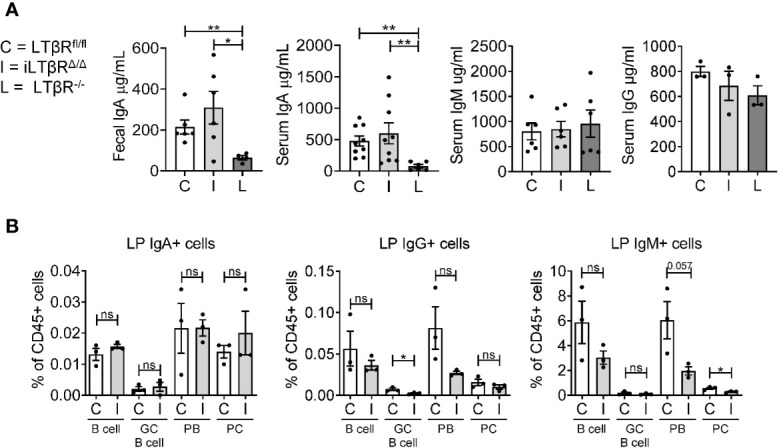
LTβR signaling in adulthood is dispensable for the IgA production. iLTβR^Δ/Δ^ (I) mice were analyzed 4 months after TAM administration. LTβR^fl/fl^ (C) and LTβR^-/-^ (L) mice were used as controls. **(A)** Total IgA, IgM and IgG levels measured by ELISA (n=3-9 mice per group). Significance determined by one-way ANOVA with Dunn’s correction. Collective data from two separate experiments is shown. **(B)** Flow cytometry analysis of immunoglobulin producing cells from colon LP: CD138^-^CD19^+^GL7^-^ B cells, CD138^-^CD19^+^GL-7^+^ germinal center (GC) B cells, CD138^+^CD19^+^ plasmablasts (PB), CD138^+^CD19^-^ plasma cells (PC). Graphs depict % of cells in CD45^+^ gate. N=3 for all groups. Significance was determined by unpaired t-test. Data shown is from one representative experiment out of two. Gating strategy shown in **Figure S5**. Data shown are means ± SEM. Not significant (ns, p > 0.05), *p < 0.05, **p < 0.01.

LTβR^-/-^ mice display severely reduced levels of IgA in the gut and blood (16). Since LTβR^-/-^ mice have developmental defects in the formation of gut-associated lymphoid organs, it is not clear whether LTβR signaling is required for IgA production in adulthood. To define the impact of LTβR inactivation in adulthood for IgA production, we measured fecal and serum IgA levels in adult iLTβR^Δ/Δ^ mice four months after TAM treatment (**Figure 5**). We chose a four month time period for the analysis of non-specific antibody responses to allow sufficient time for *de novo* generation of IgA producing plasma cells after inactivation of LTβR. Surprisingly, IgA levels in both serum and feces of iLTβR^Δ/Δ^ mice were comparable to those of WT mice, in contrast to reduced IgA levels in LTβR^-/-^ mice (**Figure 5A**). IgG and IgM levels were not affected in both iLTβR^Δ/Δ^ and LTβR^-/-^ mice as expected (**Figure 5A**). Accordingly, we did not find difference in IgA producing cells between iLTβR^Δ/Δ^ mice and LTβR^fl/fl^ control mice (**Figure 5B** and **Figure S5**). Examination of IgG producing cells revealed a trend toward decreased frequency of IgG^+^ plasmablasts (PB) as well IgG^+^ GC B cells (**Figure 5B** center panel) in iLTβR^Δ/Δ^ mice. IgM^+^ plasma cells (PC), PB, and B cells were slightly reduced in iLTβR^Δ/Δ^ mice, albeit not significantly (**Figure 5B**). Thus, these results suggest that inactivation of LTβR signaling in adulthood does not result in impaired maintenance of IgA production.

### LTβR Signaling Is Required for *De Novo* IgA Production and Protection Against Mucosal Bacterial Pathogen

**Figure 6:**
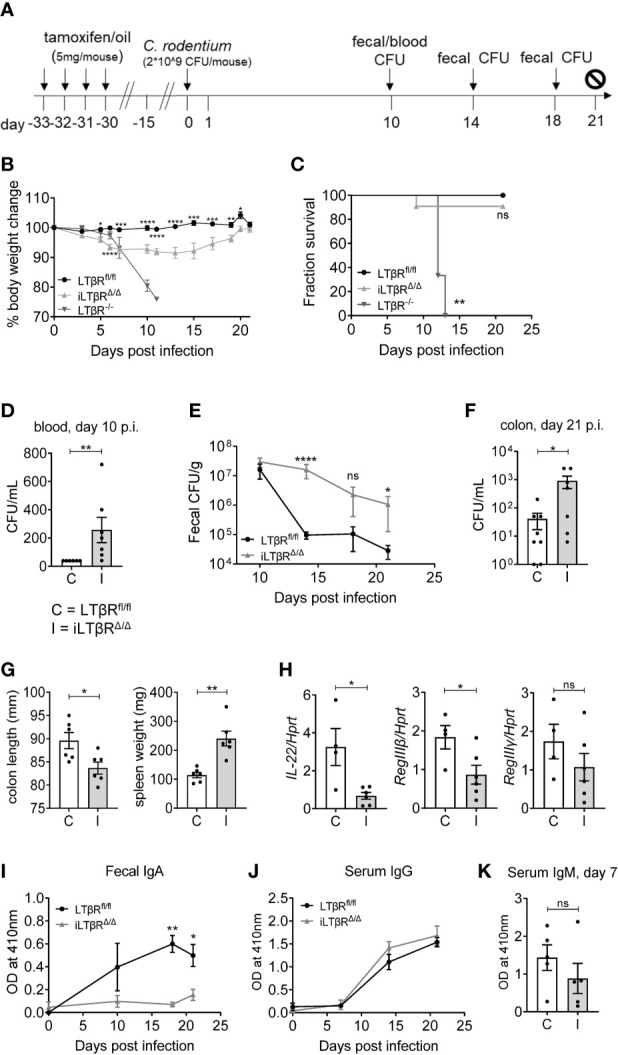
LTβR signaling contributes to protection against *C. rodentium* and is required for the generation of *C. rodentium*-specific IgA responses. **(A)** Scheme for *C. rodentium* infection and TAM treatment. **(B)** Percentage body weight change over the course of infection. **(C)** Analysis of survival. Significance was determined using the Log-rank (Mantel-Cox) and Gehan-Breslow-Wilcoxon tests. **(D–F)** Bacterial titers in blood **(D)**, feces **(E)** and colons **(F)** at the indicated timepoints. **(G)** Colon length and spleen weight at 21d post infection. **(H)** Expression of IL-22, RegIIIβ and RegIIIγ in the colon determined by qPCR at 6d post infection. **(I, J)** Analysis of *C. rodentium*-specific fecal IgA **(I)**, serum IgG **(J)**, and serum IgM **(K)** levels by ELISA. Results shown are representative figures from three independent experiments (n=3-11 mice per group). Significance for panels B, E, I, and J were determined by two-way ANOVA followed by Mann-Whitney tests. For panels D, F, G, H and K significance was determined using Mann-Whitney tests. Data shown are means ± SEM. Bars show the mean, symbols represent individual mice. Not significant (ns, p > 0.05), *p < 0.05, **p < 0.01, ***p < 0.001, ****p < 0.0001.

To further define the impact of LTβR signaling in mucosal immune responses, we orally infected iLTβR^Δ/Δ^ mice with *C. rodentium* (**Figure 6A**). *C. rodentium* is a murine pathogen which mimics the diarrheagenic disease caused by the human pathogens enteropathogenic *Escherichia coli* (EPEC) and enterohemorrhagic *E coli* (EHEC) (44, 64). LTβR^-/-^ mice are highly sensitive to *C. rodentium* infection, which results in 100% mortality in these mice (65, 66). iLTβR^Δ/Δ^ mice showed an increased susceptibility to *C. rodentium* infection, as they exhibited increased body weight loss (**Figure 6B**), colon shortening (**Figure 6G**), increased spleen weight (**Figure 6G**), and increased bacterial titers in their blood and colons (**Figures 6D–F**) compared to LTβR^fl/fl^ control mice. However, 90% of iLTβR^Δ/Δ^ mice survived infection, compared to 100% mortality in LTβR^-/-^ mice (**Figure 6C**). Multiple immune abnormalities and lack of gut-associated lymphoid tissues in LTβR^-/-^ mice could be responsible for their increased susceptibility, compared to iLTβR^Δ/Δ^ mice. Rapid production of IL-22 after *C. rodentium* infection is one of the major mechanisms of protection against this pathogen (64). Impaired IL-22 production in the colon of LTβR^-/-^ mice leads to increased susceptibility of these mice to *C. rodentium* (67, 68). Expression of IL-22 and IL-22-dependent antibacterial protein RegIIIβ were significantly reduced in colon of iLTβR^Δ/Δ^ mice compared to LTβR^fl/fl^ control mice, whereas RegIIIγ was not notably affected (**Figure 6H**).

We next thought to define the impact of LTβR inactivation on generation of *C. rodentium*-specific antibody responses (**Figures 6I–K**). We found reduced fecal levels of *C. rodentium*-specific IgA in iLTβR^Δ/Δ^ mice, whereas serum IgG and IgM were not changed compared to LTβR^fl/fl^ mice (**Figures 6I–K**). These results suggest that disruption of LTβR signaling in adulthood increases susceptibility to *C. rodentium* infection and blocks generation of pathogen-specific IgA responses.

## Discussion

LTβR is known as key regulator of lymphoid organogenesis. Nearly three decades of work has expanded the role of LTβR beyond the scope of lymphoid organ development and maintenance to include roles the in regulation of mucosal repair, cancer, inflammation and autoimmunity (6, 25, 41, 66–69). LTβR^-/-^ mice provided a useful animal model to address the role of LTβR signaling *in vivo*. However, to better understand the role of LTβR signaling in the maintenance of lymphoid organs and immune homeostasis in adulthood, we need to uncouple these functions from the developmental defects and systemic inflammation observed in LTβR^-/-^ mice. In this study we redefined the role of LTβR signaling in adulthood using mice with inducible genetic inactivation of LTβR. Though a potential issue with all conditional gene targeted strategies is efficacy of gene deletion, we demonstrated strong gene deletion in several tissues including colon, liver, kidney, and LNs. Our results suggest that LTβR signaling in adulthood is critical for the maintenance of spleen, lymph nodes, and gut-associated lymphoid organs; homeostasis of neutrophils, NK and iNKT cells; and specific IgA antibody responses against mucosal pathogen *Citrobacter rodentium*. Furthermore, our results indicate that adult stage LTβR signaling is dispensable for the maintenance of polyclonal IgA responses and autoimmunity.

Analysis of LNs in iLTβR^Δ/Δ^ mice helped us clarify the role of LTβR signaling during adulthood in regulating the cellularity and microarchitecture of LNs. Our results demonstrate that active LTβR signaling is required for the migration of B, CD4^+^ T and CD8^+^ T cells to the LNs. Reduced cellularity of LNs was also observed in a previous study using long-term 28 day biochemical inhibition of LTβ and LIGHT signaling with LTβR-Ig fusion protein in BALB/C mice (31). In contrast, other studies showed that short-term LTβR-Ig administration in adult mice or in neonate mice did not affect LN size (18, 70), suggesting that long-term and short-term inhibition of LTβR signaling have different impacts on LN cellularity. Reduced LN cellularity was consistent with reduced expression of CXCL13, CCL19, and CCL21 chemokines. In fact, ablation of LTβR specifically in CXCL13 expressing cells (using CXCL13-Cre) reduced cellularity of LNs and ablated formation of B cell follicles without affecting the development of LNs during embryogenesis (71). Ablation of LTβR in endothelial cells resulted in impaired formation of LNs during embryogenesis and reduced cellularity in adult mice (71, 72). In contrast, targeting LTβR in CCL19-expressing cells (CCL19-Cre) did not affect LN development and structure, but impaired resistance to viral infection (73). Thus, LTβR signaling in distinct cell populations is responsible for the development, cellularity, and microarchitecture of LNs. It remains to be determined whether impaired microarchitecture of LNs in iLTβR^Δ/Δ^ mice would result in increased susceptibility to pathogens.

Further analysis of LN microarchitecture in iLTβR^Δ/Δ^ mice revealed reduction of FDCs, disruption of B cell follicles and disorganization of marginal sinus macrophages. FDC survival is known to be highly dependent on LTβR signaling, as LTβR-Ig treatment resulted in rapid elimination of FDCs (within 24h) in the LNs and spleen (74). In contrast, prolonged LTβR inhibition (for several weeks) is required for disruption of B cell follicles, sinus macrophages in lymph nodes and the marginal zone of the spleen (49, 70, 75). Consistent with these previous observations, we found a reduction of CD169^+^ SCS macrophages in the MLN of iLTβR^Δ/Δ^ mice. However, CD169^+^SCS macrophages co-expressed SIGNR1, which is normally expressed by medulla macrophages (48). Similar co-expression of CD169 and SIGNR1 by SCS macrophages has been observed in mice treated with LTβR-Ig (75). It remains to be determined whether CD169^+^ SCS macrophages were replaced with CD169^+/lo^SIGNR1^+^ medullary sinus macrophages or converted their phenotype in iLTβR^Δ/Δ^ mice. LT provided by B cells was shown to be responsible for the maintenance of SCS macrophages (22, 75). Conversely, the cellular source for the maintenance of FDCs in LNs remains unclear, as elimination of LT from B cells only resulted in minimal disruption of LN FDCs (22). It is possible that cooperation of LT signaling by B cells, T cells, and ILCs is necessary to support the maintenance of FDCs in LNs.

Our analysis of PPs and ILFs in iLTβR^Δ/Δ^ mice is in agreement with previous studies which used long-term biochemical inhibition of LTβR (LTβR-Ig) in adult mice (53). Specifically, LTβR blockade was associated with flattened PP appearance and reduction in the number of macroscopically visible PPs (76) and ILFs (53). Additionally, we found that active LTβR signaling in adulthood is required for the formation of FDCs in PPs. The function of FDCs in PPs was previously associated with generation of IgA in the gut (77). However, our study did not find defects in polyclonal IgA production in iLTβR^Δ/Δ^ mice. In contrast, specific IgA responses to enteric pathogen *C. rodentium* were reduced in iLTβR^Δ/Δ^ mice.

We next analyzed the impact of inducible LTβR inactivation on spleen microarchitecture in adult mice. Our analysis revealed impaired formation of B cell follicles and reduced FDCs in the spleen. These defects were consistent with biochemical inhibition of LTβR using LTβR-Ig reagent (70, 74), supporting the role of LTβR signaling in the maintenance of B cell follicles and FDCs in the spleen. The integrity of the marginal zone was also impaired in iLTβR^Δ/Δ^ mice as evidenced by reduced MAdCAM-1^+^ sinus endothelial cells, CD169^+^ metallophilic macrophages, and SIGNR1^+^ macrophages. In contrast, MZ structure is completely ablated in LTβR^-/-^ mice (28) as formation of MAdCAM-1^+^ marginal sinus is fixed in the early postnatal period and cannot be restored in adult mice by transplantation of bone marrow from WT mice (19). Our results are consistent with biochemical inhibition of LTβR signaling in adult mice, which demonstrated various degrees of disruption of MAdCAM-1^+^ sinus lining cells, marginal zone CD169^+^ metallophilic macrophages and SIGNR1^+^ marginal zone macrophages depending on the dose and duration of treatment (19, 70). B cells are the primary source of LT required for the maintenance of FDCs and support of marginal zone integrity (22), with additional help from LT-expressing T cells (78). Although LT expressed by ILC3 contributes to early postnatal development of white pulp (79, 80), its role for the maintenance of spleen microarchitecture in adult mice is less clear. Inactivation of LT on ILC3s did not result in defects in the formation of FDCs and B cell follicles in adult mice, whereas differentiation of cDC2 in spleen was impaired (81). In contrast to homeostatic conditions, LT from ILC3s can contribute to the restoration of spleen microarchitecture after viral-induced tissue damage (82). Likewise, LIGHT^-/-^ mice do not display defects in organization of spleen and lymph nodes in homeostatic conditions (83). However, LIGHT signaling *via* LTβR can restore FDCs and B cell follicles in LT-deficient mice (23) as well as support LN remodeling during inflammatory response (24). Thus, we propose that the requirements for LTβR signaling in the maintenance of lymphoid organs during homeostatic conditions and during inflammation can be different and provided by distinct LT and LIGHT producing cells. iLTβR^Δ/Δ^ mice can be used as a robust model to test this hypothesis in follow up studies.

LTβR^-/-^ mice develop an autoimmune phenotype such as splenomegaly, production of autoantibodies and lymphocytic infiltrations to non-lymphoid organs (28–30, 57, 84, 85). The mechanistic explanation behind an autoimmune phenotype in LTβR-deficient mice remains a contentious topic. Since an autoimmune phenotype in LTβR^-/-^ mice can be due to developmental defects in lymphoid organs and intestinal microbiota composition (28, 30, 32, 36, 56, 57), the role of LTβR signaling in autoimmunity and systemic inflammation in adulthood remains controversial. We assessed key parameters which previously have been associated with systemic inflammation and autoimmunity in LTβR^-/-^ mice. Our results demonstrate that inactivation of LTβR signaling during adulthood in iLTβR^Δ/Δ^ mice did not result in splenomegaly, infiltration of immune cells to non-lymphoid organs, impairment of thymus microarchitecture, or reduction of thymic *Aire* expression. We also did not find abnormal production of dsDNA autoantibodies in iLTβR^Δ/Δ^ mice. Thus, we conclude that inhibition of LTβR signaling in adulthood does not lead to systemic inflammation and autoimmunity. These results are important to consider in translational studies, as agonists and antagonists of LTβR signaling are currently being tested as potential therapies in autoimmune diseases and cancer (5, 6, 69, 86, 87).

Our analysis of innate immune cell populations revealed a reduced number of neutrophils in the spleens of iLTβR^Δ/Δ^ mice. In contrast, LTβR^-/-^ mice were reported to have increased neutrophil numbers in the spleen which was dependent on microbiota as antibiotic treatment of LTβR^-/-^ mice reduced neutrophil numbers (30). Reduced frequencies of neutrophils in iLTβR^Δ/Δ^ mice was not due to differences in microbiota, as iLTβR^Δ/Δ^ mice and littermate LTβR^fl/fl^ control mice were co-housed in our experiments. Reduced neutrophil frequencies in the spleen of iLTβR^Δ/Δ^ mice was associated with impaired expression of key neutrophil-recruiting chemokine, CXCL2. In line with these results, previous studies suggested a role for LTβR signaling in control of CXCL1 and CXCL2 expression in response to mucosal bacterial pathogens (66) and suppression of metabolic activation *via* neutrophil-intrinsic LTβR signaling during colitis (59).

Previous studies using LTβR^-/-^ and LTα^-/-^ mice or WT mice treated with LTβR-Ig demonstrated loss of NK cell populations in the spleen, lung, blood and bone marrow as well as impairment of NK cell anti-tumor activities (13, 61). LT expression on RORγt^+^ ILC3s was suggested to be critical for NK cell development *via* interaction with bone marrow stromal cells (60). Our results in iLTβR^Δ/Δ^ mice are consistent with these studies and support the role of LTβR signaling in adulthood for the homeostasis of NK cells.

LTβR^-/-^ mice have impaired NKT development due to developmental defects in thymic stroma (14, 15). However, the role of adult LTβR signaling in NKT cell development is unclear as administration of LTβR-Ig in adult WT mice did not result in impaired development of NKT cells which is in contrast to LTβR-Ig blockade during embryogenesis and neonate phases (15, 88). Consistent with these studies, we did not find defects in thymus CD1d-tet^+^TCRβ^+^NK1.1^+^ iNKT cell populations in iLTβR^Δ/Δ^ mice indicating that LTβR signaling is dispensable for development of iNKT cells in adulthood. Interestingly, iNKT cell frequencies were reduced in the livers and spleens of iLTβR^Δ/Δ^ mice. The mechanism of LTβR-dependent iNKT cell recruitment to the liver remains to be determined.

Impaired IgA production in LTβR^-/-^ mice was initially attributed to stromal cell populations that provide signals to B cells for homing and antibody class switching to IgA (16). However, due to lack of gut-associated lymphoid tissues, such as PPs, MLN, ILFs and cryptopatches in LTβR^-/-^ mice, the contribution of LTβR signaling in adulthood remained undefined. Follow up studies implicated the role of ILFs in gut IgA production that were dependent on the interaction of LT expressing ILC3s with LTβR on stromal cells (89, 90). However, genetic inactivation of LTβ on ILC3s did not result in impaired IgA production in spite of impaired formation of ILFs and reduced INOS production by DCs in MLN (17). FDCs have been suggested to contribute to IgA generation in PPs (77). Additionally, a recent study indicated the role of intrinsic LTβR signaling in PP DCs for IgA generation in response to the microbiota (91). Our results suggest that LTβR signaling in adulthood is dispensable for the maintenance of fecal or serum IgA despite reduced ILF numbers and impaired FDCs in PPs. Consistently, we found very efficient LTβR deletion in the colon. These results suggest that other compensatory pathways contribute to IgA production when LTβR signaling is inhibited in adult mice. We also do not exclude the possibility of long-lived plasma cells that could survive for several months after LTβR inactivation and contribute to IgA production in iLTβR^Δ/Δ^ mice. The non-specific IgA antibodies we detected in iLTβR^Δ/Δ^ mice could be derived from such long-lived cells that underwent antibody class switching prior to inactivation of LTβR. The impact of LTβR signaling on IgA production in humans remains to be determined, as human and mouse IgA systems have distinct anatomical and functional differences, including presence of two IgA1 and IgA2 isotypes, lack of cryptopatches and TLR4 expression by B cells in humans (92).

To define the role of LTβR signaling in generation of specific IgA in response to mucosal bacterial infection, we infected iLTβR^Δ/Δ^ mice with *C. rodentium*. While iLTβR^Δ/Δ^ mice showed an increased susceptibility to *C. rodentium* infection, demonstrated by increased weight loss and increased bacterial load, this phenotype was less pronounced compared to LTβR^-/-^ mice. Importantly, we found reduced *C. rodentium*-specific IgA levels in feces of iLTβR^Δ/Δ^ mice, whereas serum IgG and IgM were not changed. These results suggest that LTβR signaling in adulthood contributes to generation of pathogen-specific IgA. Impaired structure of PPs and MLN as well as reduced numbers of ILFs could contribute to the defect in *C. rodentium* specific IgA production, consistent with the role of these tissues in response to bacterial antigens (77, 91). Interestingly, a recent study demonstrated that treatment of adult mice with LTβR-Ig did not result in impaired rotavirus-specific IgA production, whereas LTβR blockade during embryogenesis and neonate period impaired rotavirus-specific IgA production (18). These and our own results suggest distinct LTβR requirements in adulthood for production of IgA in response to bacterial and viral pathogens.

In summary, our study redefined the role of LTβR signaling in adulthood for organization of lymphoid organs, autoimmunity, homeostasis of innate immune cells and IgA production. Our results suggest that inducible genetic LTβR inactivation during adulthood results in impaired organization of LNs and spleen; homeostasis of neutrophils, NK, and iNKT cells; and generation of mucosal pathogen-specific IgA responses but does not result in autoimmunity. Mice with inducible genetic inactivation of LTβR provide a robust preclinical model to evaluate the impact of LTβR agonists and inhibitors in disease treatment.

## Data Availability Statement

The raw data supporting the conclusions of this article will be made available by the authors, without undue reservation.

## Ethics Statement

The animal study was reviewed and approved by Institutional Animal Care and Use Committee of University of Texas Health Science Center San Antonio.

## Author Contributions 

Study concept and design: EK and AT. Designed and performed experiments, analyzed data, edited manuscript: YS, EK, AM, AK, SS, AT, CS, KY, EL, RP, and AA. Drafted manuscript: YS and AM. Wrote and edited manuscript: AT and EK. All authors contributed to the article and approved the submitted version.

## Funding

This research was supported by grant from NIH (AI135574, NS112263). AT was supported by the Max and Minnie Tomerlin Voelcker Fund, William and Ella Owens Medical Research Foundation. AM was supported by K12 GM111726 San Antonio Biomedical Education and Research-Institutional Research and Academic Career Development Award (SABER-IRACDA). Flow Cytometry and Optical Imaging Core facilities at UT Health San Antonio is supported with funding from University and the NIH (NCI P30 CA054174).

## Conflict of Interest

The authors declare that the research was conducted in the absence of any commercial or financial relationships that could be construed as a potential conflict of interest.

## Supplementary Material

Supplementary Figure 1 Efficacy of inducible LTβR inactivation in iLTβR^Δ/Δ^ mice. **(A)** Analysis of LTβR expression in colon, thymus, liver, lung, kidney, spleen, mesenteric LN lymph node (MLN), inguinal LN (ILN) and Peyer`s patches (PPs) measured by comparing LTβR mRNA levels in specified tissues between LTβR^fl/fl^ (C) and iLTβR^Δ/Δ^ (I) mice by qPCR one month after TAM administration. N=4-12 mice per group. Combined data from 2-3 experiments is shown. **(B)** qPCR comparison of oral (O) and intraperitoneal (IP) administration of TAM on LTβR inhibition in the colon. LTβR^fl/fl^ mice treated with corn oil were used as controls. Data shown was from a single experiment. **(C)** Analysis of LTβR gene deletion by PCR of DNA from colons of LTβR^fl/fl^ and iLTβR^Δ/Δ^ mice. Representative images from 3 separate experiments are shown. **(D)** Location of genotyping primers in LTβR^fl/fl^ mice. Significance was determined for **(A, B)** by unpaired t-test. Not significant (ns, p > 0.05), *p < 0.05, **p < 0.01, ***p < 0.001, ****p < 0.0001.

Supplementary Figure 2 Flow cytometry gating strategy for NK, iNKT, and neutrophils. **(A)** Gating for NK, iNKT, B, and T cells in the spleen. **(B)** Gating for neutrophils in the spleen. **(C)** Gating strategy for T, B, NK, and iNKT cells in thymus.

Supplementary Figure 3 Distribution of tissue specific B cell, CD4^+^ and CD8^+^ T cell populations by flow cytometry. Evaluation of B and T cell populations in LTβR^fl/fl^ (C) and LTβR^-/-^ (L) mice 2 months after TAM treatment in MLN **(A)**, ILN **(B)**, PPs **(C)**, spleen **(D)**, blood **(E)**, and thymus **(F)**. % of cells among CD45^+^ cells and total cell numbers are shown. Data are combined from 3 experiments for panels **(A, D, E)** Representative data from two experiments is shown for panels **(B, F)** Panel **(C)** shows representative data from one of two experiments. N=3-14 for each group. Significance was determined by Mann-Whitney test or one-way ANOVA with Tukey’s correction for multiple comparisons. Data shown are means ± SEM. Bars show the mean, symbols represent individual mice. Not significant (ns, p >0.05), *p < 0.05, **p < 0.01.

Supplementary Figure 4 Histological analysis of iLTβR^Δ/Δ^ mice. **(A)** Representative H&E staining of formalin-fixed sections from LTβR^fl/fl^ (C), iLTβR^Δ/Δ^ (I), and LTβR^-/-^ (L) mice 2 months after TAM treatment. Scale bars = 100µm (ILF), 200µm (colon and spleen), or 800µm (small intestine, SI). N=5 mice per genotype. **(B)** Quantification of isolated lymphoid follicles (ILF) in the colon and SI. Data shown is the average number of ILFs per mouse for a single experiment with n=2-5 per group. Significance was determined by Mann-Whitney test. **(C)** Spleen weight. Collective data from 3 experiments shown (n=3-12 per group). Significance was determined by Kruskal Wallis test with Dunn’s correction followed by Mann-Whitney test to compare groups I and L. **(D)** Impaired FDCs and marginal zone in iLTβR^Δ/Δ^ mice. Frozen spleen sections were stained with CR1 and MAdCAM-1 antibodies followed by secondary HRP-conjugated anti-rat antibody. Scale bars = 200µm. Representative images are shown (n=4 per group). *p < 0.05, **p < 0.01.

Supplementary Figure 5 Flow cytometry gating strategy for IgA, IgM, and IgG expressing B cells. Gating strategy for the determination of IgG, IgM, IgA expressing cells within B cell populations from the colon lamina propria. Populations were defined as: CD138^-^CD19^+^GL7^-^ B cells, CD138^-^CD19^+^GL-7^+^ germinal center (GC) B cells, CD138^+^CD19^+^ plasmablasts (PB), CD138^+^CD19^-^ plasma cells (PC).

## Abbreviations

LTβR, lymphotoxin beta receptor; LT, lymphotoxin; LIGHT, lymphotoxin-like inducible protein that competes with glycoprotein D for herpes virus entry on T cells; HVEM, herpes virus entry mediator; TAM, tamoxifen; *C. rodentium*, *Citrobacter rodentium*; LN, lymph node; MLN, mesenteric LN; ILN, inguinal LN; PP, Peyer’s patches; ILF, isolated lymphoid follicle; SI, small intestine; WT, wild type; FDC, follicular dendritic cell; ILC, innate lymphoid cell; SCS, subcapsular sinus.
