# Supplementary figures and images for: Redefining the Role of Lymphotoxin Beta Receptor in the Maintenance of Lymphoid Organs and Immune Cell Homeostasis in Adulthood
Source: Front Immunol. 2021 Jul 15;12:712632. doi: 10.3389/fimmu.2021.712632 (PMC8320848; doi:10.3389/fimmu.2021.712632)

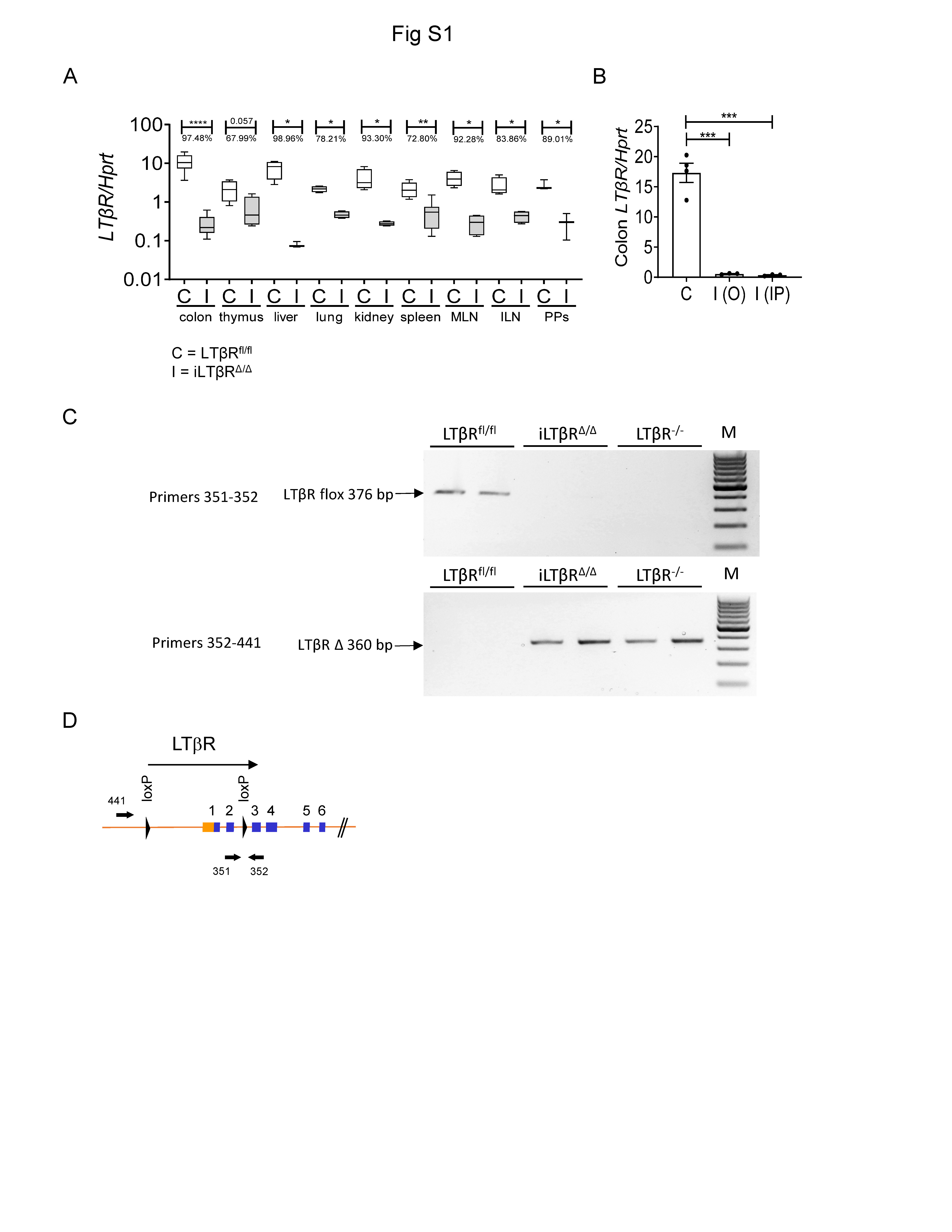

Supplement: Supplementary Figure 1 — Efficacy of inducible LTβR inactivation in iLTβRΔ/Δ mice. (A) Analysis of LTβR expression in colon, thymus, liver, lung, kidney, spleen, mesenteric LN lymph node (MLN), inguinal LN (ILN) and Peyer`s patches (PPs) measured by comparing LTβR mRNA levels in specified tissues between LTβRfl/fl (C) and iLTβRΔ/Δ (I) mice by qPCR one month after TAM administration. N=4-12 mice per group. Combined data from 2-3 experiments is shown. (B) qPCR comparison of oral (O) and intraperitoneal (IP) administration of TAM on LTβR inhibition in the colon. LTβRfl/fl mice treated with corn oil were used as controls. Data shown was from a single experiment. (C) Analysis of LTβR gene deletion by PCR of DNA from colons of LTβRfl/fl and iLTβRΔ/Δ mice. Representative images from 3 separate experiments are shown. (D) Location of genotyping primers in LTβRfl/fl mice. Significance was determined for (A, B) by unpaired t-test. Not significant (ns, p > 0.05), *p < 0.05, **p < 0.01, ***p < 0.001, ****p < 0.0001. [file Image_1.jpeg]

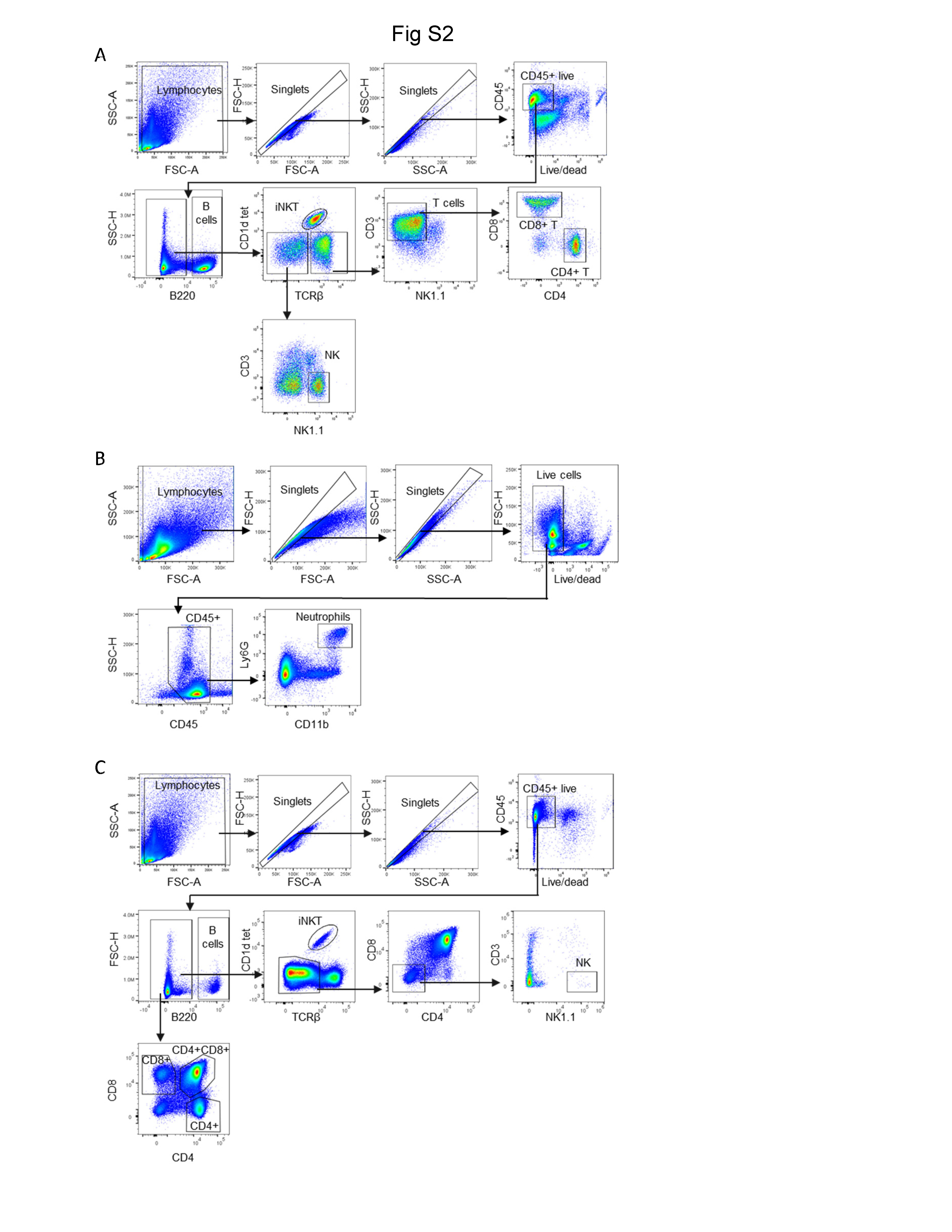

Supplement: Supplementary Figure 2 — Flow cytometry gating strategy for NK, iNKT, and neutrophils. (A) Gating for NK, iNKT, B, and T cells in the spleen. (B) Gating for neutrophils in the spleen. (C) Gating strategy for T, B, NK, and iNKT cells in thymus. [file Image_2.jpeg]

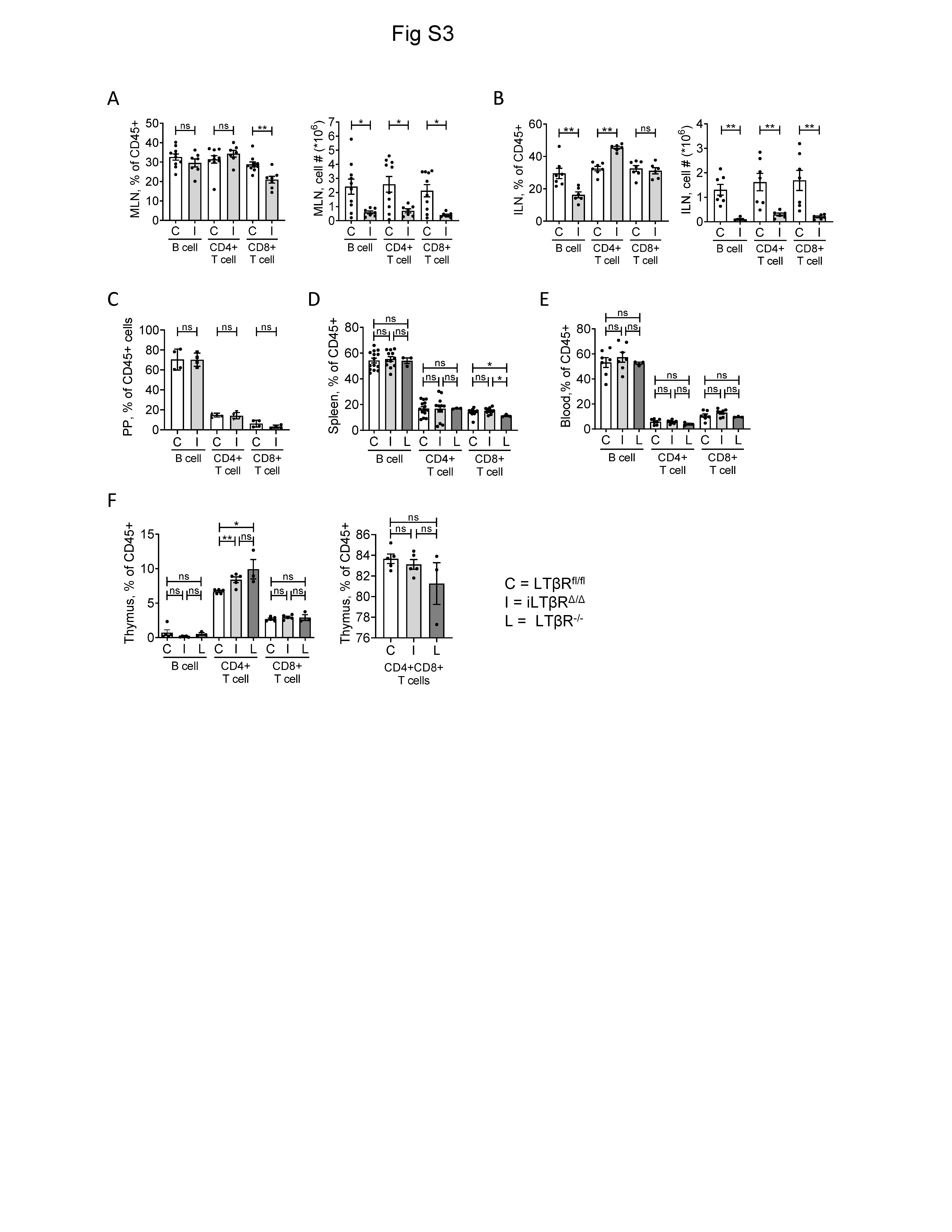

Supplement: Supplementary Figure 3 — Distribution of tissue specific B cell, CD4+ and CD8+ T cell populations by flow cytometry. Evaluation of B and T cell populations in LTβRfl/fl (C) and LTβR-/- (L) mice 2 months after TAM treatment in MLN (A), ILN (B), PPs (C), spleen (D), blood (E), and thymus (F). % of cells among CD45+ cells and total cell numbers are shown. Data are combined from 3 experiments for panels (A, D, E) Representative data from two experiments is shown for panels (B, F) Panel (C) shows representative data from one of two experiments. N=3-14 for each group. Significance was determined by Mann-Whitney test or one-way ANOVA with Tukey’s correction for multiple comparisons. Data shown are means ± SEM. Bars show the mean, symbols represent individual mice. Not significant (ns, p >0.05), *p < 0.05, **p < 0.01. [file Image_3.jpeg]

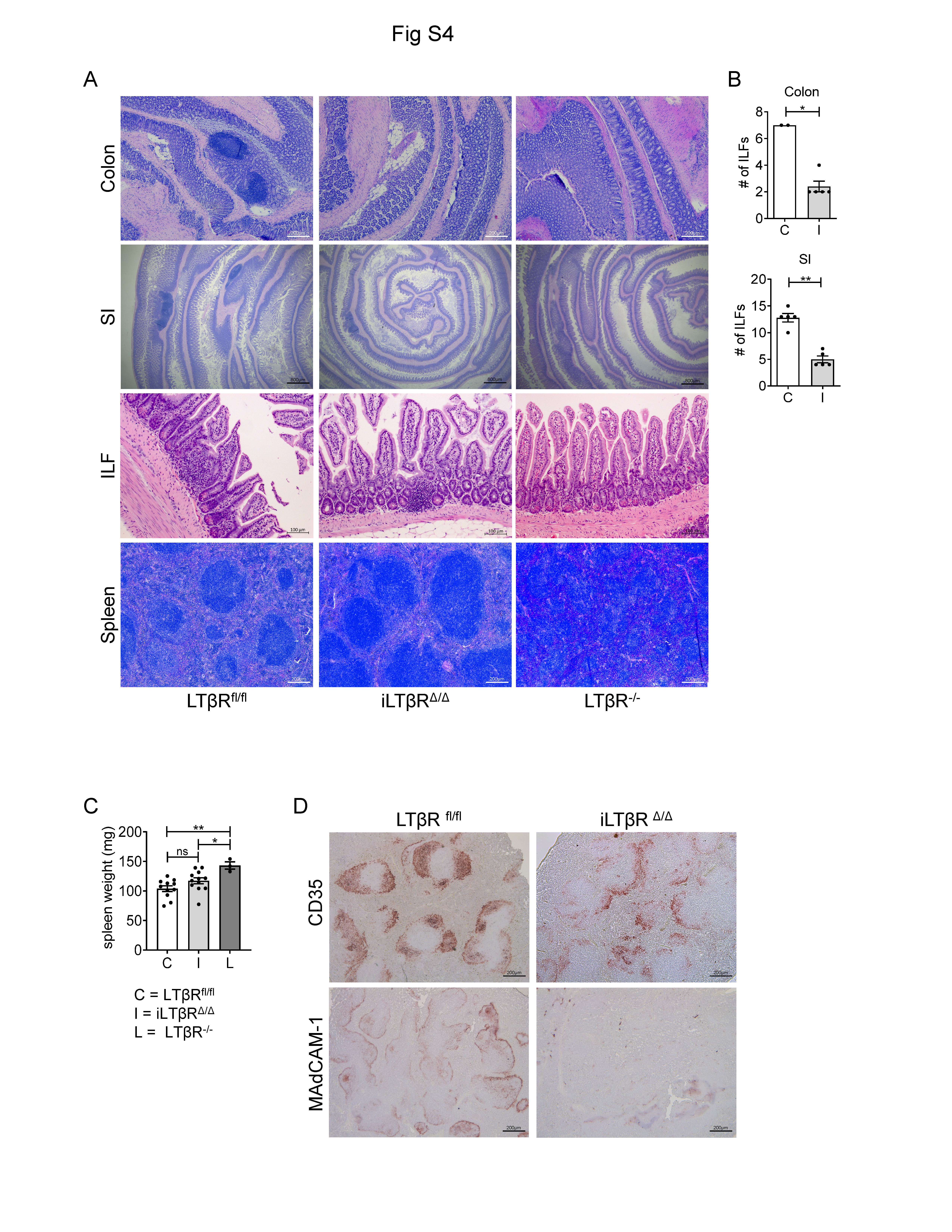

Supplement: Supplementary Figure 4 — Histological analysis of iLTβRΔ/Δ mice. (A) Representative H&E staining of formalin-fixed sections from LTβRfl/fl (C), iLTβRΔ/Δ (I), and LTβR-/- (L) mice 2 months after TAM treatment. Scale bars = 100µm (ILF), 200µm (colon and spleen), or 800µm (small intestine, SI). N=5 mice per genotype. (B) Quantification of isolated lymphoid follicles (ILF) in the colon and SI. Data shown is the average number of ILFs per mouse for a single experiment with n=2-5 per group. Significance was determined by Mann-Whitney test. (C) Spleen weight. Collective data from 3 experiments shown (n=3-12 per group). Significance was determined by Kruskal Wallis test with Dunn’s correction followed by Mann-Whitney test to compare groups I and L. (D) Impaired FDCs and marginal zone in iLTβRΔ/Δ mice. Frozen spleen sections were stained with CR1 and MAdCAM-1 antibodies followed by secondary HRP-conjugated anti-rat antibody. Scale bars = 200µm. Representative images are shown (n=4 per group). *p < 0.05, **p < 0.01. [file Image_4.jpeg]

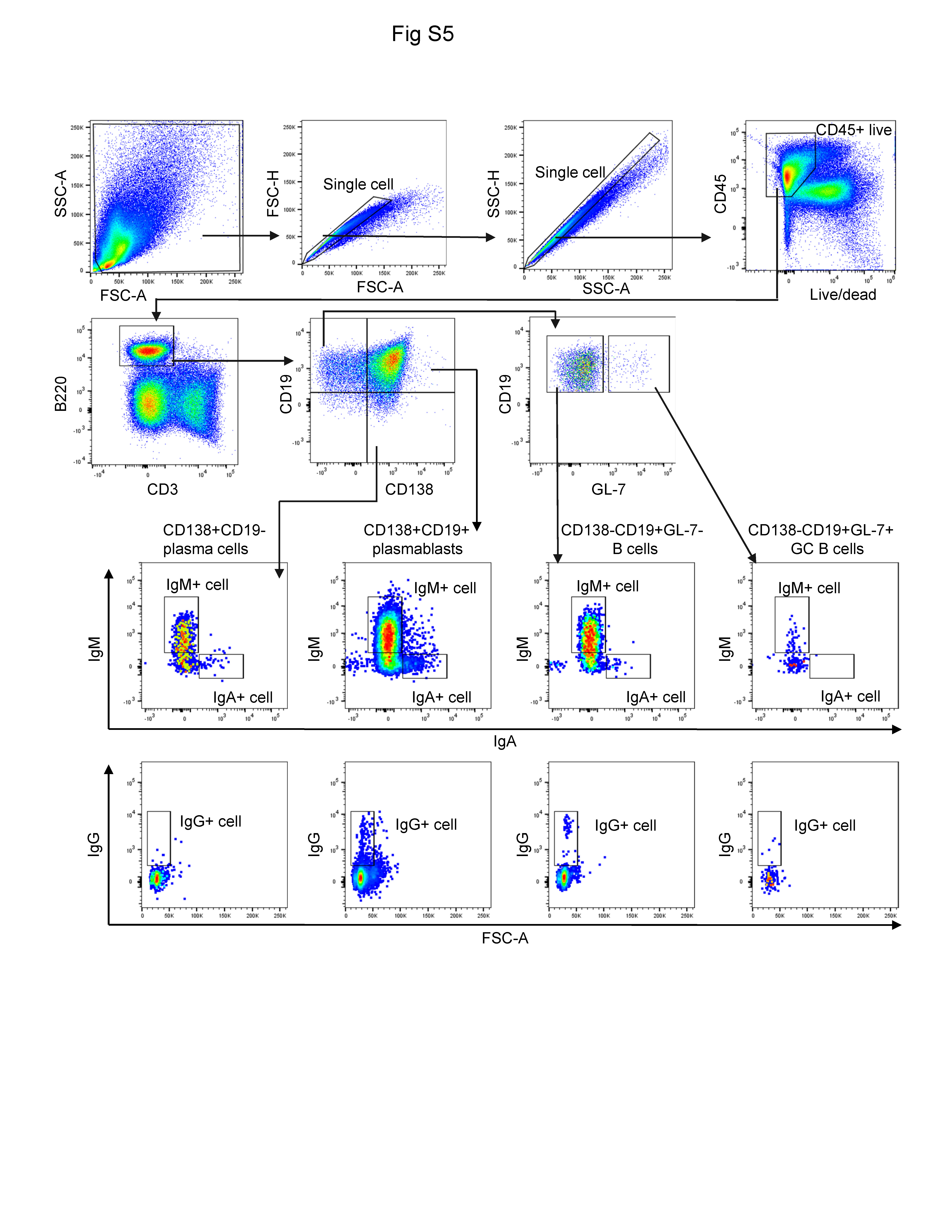

Supplement: Supplementary Figure 5 — Flow cytometry gating strategy for IgA, IgM, and IgG expressing B cells. Gating strategy for the determination of IgG, IgM, IgA expressing cells within B cell populations from the colon lamina propria. Populations were defined as: CD138-CD19+GL7- B cells, CD138-CD19+GL-7+ germinal center (GC) B cells, CD138+CD19+ plasmablasts (PB), CD138+CD19- plasma cells (PC). [file Image_5.jpeg]
