# Supplementary material for: Redefining the Role of Lymphotoxin Beta Receptor in the Maintenance of Lymphoid Organs and Immune Cell Homeostasis in Adulthood
Source: Front Immunol. 2021 Jul 15;12:712632. doi: 10.3389/fimmu.2021.712632 (PMC8320848; doi:10.3389/fimmu.2021.712632)
Supplement: Supplementary file 6 [file Table_1.docx]

**Supplementary Table 1. Primers for quantitative PCR**

| Name | Forward primer | Reverse primer |
| --- | --- | --- |
| Hprt | GCGTCGTGATTAGTGATGATGAAC | GAGCAAGTCTTTTCAGTCCTGTCCA |
| IL-22 | TCCGAGGAGTCAGTGCTAAA | AGAACGTCTTCCAGGGTGAA |
| LTβR | GCTCCAGGTACCTCCTACTCG | ATGGCCAGCAGTAGATTG |
| CXCL1 | GCTGGGATTCACCTCAAGAA | TGGGGACACCTTTTAGCATC |
| CXCL2 | CCTGGTTCAGAAAATCATCCA | CTTCCGTTGAGGGACAGC |
| CXCL12 | CTGCATCAGTGACGGTAAACC | CAGCCGTGCAACAATCTGAA |
| RegIIIβ | ATGGCTCCTACTGCTATGCC | GTGTCCTCCAGGCCTCTTT |
| RegIIIγ | ATGGCTCCTATTGCTATGCC | GATGTCCTGAGGGCCTCTT |
| CXCL13 | CAGAATGAGGCTCAGCACAGC | CAGAATACCGTGGCCTGGAG |
| CCL19 | TGTGTTCACCACACTAAGGGG | CCTTTGTTCTTGGCAGAAGACT |
| CCL21 | ATCCCGGCAATCCTGTTCTC | GGGGCTTTGTTTCCCTGGG |
| Aire | GGTTCTGTTGGACTCTGCCCTG | TGTGCCACGACGGAGGTGAG |
